# Deep graph neural network-based prediction of acute suicidal ideation in young adults

**DOI:** 10.1038/s41598-021-95102-7

**Published:** 2021-08-04

**Authors:** Kyu Sung Choi, Sunghwan Kim, Byung-Hoon Kim, Hong Jin Jeon, Jong-Hoon Kim, Joon Hwan Jang, Bumseok Jeong

**Affiliations:** 1 Graduate School of Medical Science and Engineering, Korea Advanced Institute for Science and Technology (KAIST), 291 Daehak-ro, Yuseong-gu, Daejeon, 34141 Republic of Korea; 2 Department of Psychiatry, Yonsei University College of Medicine, Seoul, Republic of Korea; 3 Department of Bio and Brain Engineering, Korea Advanced Institute for Science and Technology (KAIST), Daejeon, Republic of Korea; 4 Department of Psychiatry, Depression Center, Samsung Medical Center, Sungkyunkwan University School of Medicine, Seoul, Republic of Korea; 5 Department of Psychiatry, Gil Medical Center, Gachon University College of Medicine, Gachon University, Incheon, Republic of Korea; 6 Neuroscience Research Institute, Gachon Advanced Institute for Health Science and Technology, Gachon University, Incheon, Republic of Korea; 7 Department of Human Systems Medicine, Seoul National University College of Medicine, 103 Daehak-ro, Jongro-gu, Seoul, 03080 Republic of Korea; 8 KAIST Institute for Health Science and Technology, Korea Advanced Institute for Science and Technology (KAIST), Daejeon, Republic of Korea; 9 KAIST Clinic Pappalardo Center, Korea Advanced Institute for Science and Technology (KAIST), Daejeon, Republic of Korea

**Keywords:** Depression, Predictive markers, Diagnosis

## Abstract

Precise remote evaluation of both suicide risk and psychiatric disorders is critical for suicide prevention as well as for psychiatric well-being. Using questionnaires is an alternative to labor-intensive diagnostic interviews in a large general population, but previous models for predicting suicide attempts suffered from low sensitivity. We developed and validated a deep graph neural network model that increased the prediction sensitivity of suicide risk in young adults (n = 17,482 for training; n = 14,238 for testing) using multi-dimensional questionnaires and suicidal ideation within 2 weeks as the prediction target. The best model achieved a sensitivity of 76.3%, specificity of 83.4%, and an area under curve of 0.878 (95% confidence interval, 0.855–0.899). We demonstrated that multi-dimensional deep features covering depression, anxiety, resilience, self-esteem, and clinico-demographic information contribute to the prediction of suicidal ideation. Our model might be useful for the remote evaluation of suicide risk in the general population of young adults for specific situations such as the COVID-19 pandemic.

## Introduction

Suicide is the second leading cause of death in young adults (individuals 10–34 years old) in the US and is 2.5 times more frequent than homicides (48,344 vs. 18,830, respectively){Xu, 2020 #29;Wu, 2020 #28}. The total suicide rate in the US in 2018 represented an increase of 35% during the previous two decades^1^. Suicidal ideation (SI) and suicide attempts (SAs), which are strong risk factors for completed suicide, are prevalent in the population (11–14% and 2.8–4.6%, respectively)^2^. Worldwide, the number of suicides is over 800,000 annually^3^, and 60–70% of suicides die on the first or “index” attempt. Additionally, only approximately 30–40% of survivors received emergent hospital-level care^4,5^. Thus, accurate prediction of first SAs, or individuals with imminent suicide risk, followed by instantaneous intervention, would be effective in suicide prevention, leading to decreased mortality in young adults.

During pandemics such as the novel coronavirus disease 2019 (COVID-19) pandemic, remote mental health evaluation of self-isolating people to prevent viral spread is critical. The monthly suicidal rate increased by 16% in Japan during the second wave of the COVID-19 pandemic^6^. Symptoms of anxiety and depressive disorder markedly increased in the US during April-June 2020^7^ compared with the same period in 2019^8^. Pre-existing psychiatric disorders were associated with increased SI as a psychological impact of the COVID-19 pandemic^9^ and those disorders contributed to predicting future individuals with SI in young adult populations^10^. Moreover, younger adults reported having experienced disproportionately worse mental health outcomes and elevated SI than older adults^11^. Thus, the development of precise remote evaluation techniques of both suicide risk and psychiatric disorders is critical for suicidal prevention as well as psychiatric well-being.

However, there are many challenges involved in evaluating suicide risk in a large general population. In a pandemic situation, it is too labor intensive and clinician dependent to conduct structured interviews or scales for SI^12^ to assess present and past mental health in an entire population. Moreover, there is a possibility of missing cases during screening with simple questionnaires in general population studies because most studies further evaluate cases only when they respond that they have SI, which could mask true patients at risk for suicide^13^. Existing prediction models^5,14–16^ for suicidal behavior achieved an accuracy over 80%, but at the same time, these models had very low sensitivity, which is due to the low incidence of SAs in the general population. For example, ~ 0.12% among a total of 19,961,059 primary care or specialty mental health visits were identified as suicide attempts^17^. The ideal model should have a balance between specificity and sensitivity, which is important for sensitively detecting individuals at risk of suicide attempts and preventing adverse side effects for individuals who are erroneously identified as at-risk, such as unnecessary hospitalization. Thus, expanding the range of suicide risk to focus on SI instead of SAs can contribute to the development of prediction models that could mitigate the class imbalance problem and balance specificity and sensitivity.

**Figure 1:**
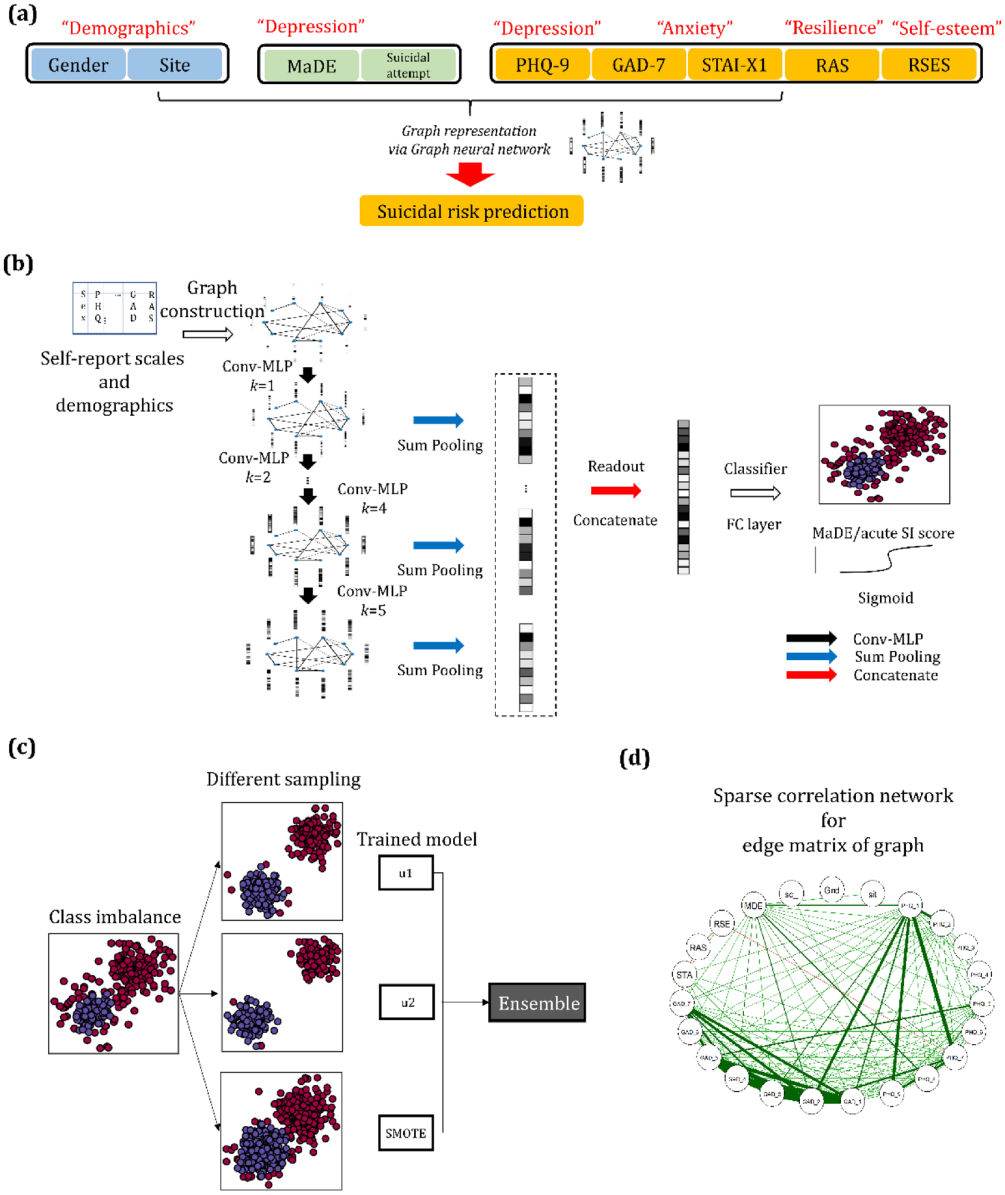
Overall architecture of a prediction model based on a graph isomorphism network (GIN): (**a**) Overall workflow for constructing the graph-structured dataset. A single subject corresponds to a single graph. (**b**) Graph constructed on multi-dimensional self-report questionnaires and clinico-demographic information, which were used as input node features and fed into five graph convolutional layers combined with MLP layers. In each layer, the graph representation is obtained after graph pooling, concatenated into a latent feature vector, and fed into a classifier using a fully connected layer to output a sigmoid prediction of MaDEs or acute SI score (0–1). (**c**) Three different GIN models with different subsampling strategies (i.e., GIN-SMOTE, GIN-u1, and GIN-u2) were ensembled to obtain the final model to overcome class imbalance. (**d**) Sparse correlation network for the edge matrix of the graph: pairwise correlation coefficients were obtained between the categorical variables of each node, representing each questionnaire item and subject information feature and were used as an edge matrix to construct a graph. All subjects share the same edge matrix to construct a graph. Note that PHQ-9 and GAD-7 show positive intra- and inter-correlations, whereas the RAS and RSES total scores are negatively correlated with the STAI-S total score. The sparsity of the graph edges was controlled by setting the threshold to 0.6. *Note*: Thickness of lines indicates degree of correlation coefficients in (**d**): i.e., a strong correlation between nodes is indicated by a thick line connecting the nodes. *MaDE* major depressive episode, *SI* suicidal ideation, *SMOTE* synthetic minority oversampling technique (u1 and u2) and under-sampling strategy (1 and 2).

In many countries, including South Korea, a large population of young adults is obliged to have regular check-ups, including mental status examinations, for work or when entering a dormitory for college, which leads to the only portal to access individuals who may attempt suicide. We employed the multiple scales included in regular mental status examinations to predict imminent suicide risk. We used acute SI within 2 weeks as a surrogate marker for prospective imminent suicide risk^18^. The definition of acute SI was an individual’s response of, “Yes” or “No”, to the question “Have you ever thought of suicide in the past 2 weeks?” in both training (n = 17,482) and test (n = 13,408) sets. To extract a good representation of acute SI from scales, multi-dimensional questionnaires evaluating depression, anxiety, resilience, and self-esteem levels were used as features for input to the neural network (Fig. 1).

Deep learning algorithms with multiple processing layers such as convolutional neural networks (CNNs) learn representations of data with multiple layers of abstraction^19^. Thus, by providing enough training data, deep learning may achieve better performance than conventional machine learning or predefined criteria-based tools^20^. In a previous study using a deep neural network model with self-report questionnaires^21^, 63.6% of participants were predicted as being at high risk for depressive disorder when they visited a psychiatric clinic as depressive disorder compared to 33.3% in a cut-off screening method. Features of an image (e.g., lines, edges, intensity), the CNN detects are based on physical distance. However, Bayesian network analyses studies showing transdiagnostic relationships suggested that the relationship among items in multi-dimensional questionnaires refers to non-Euclidean distance^10,22,23^. To overcome these challenges, we adopted the graph isomorphism network (GIN)^24^, a variant of a spatial graph neural network (GNN) specifically used for graph classification. This GIN extracts a better representation from the graph than graph convolutional networks (GCNs) do. Furthermore, to address the severe class imbalance, three kinds of data augmentation approach and their ensemble voting were introduced in the present study. To validate whether the trained model could provide information about suicidal risk as well as prediction of SI, correlation analyses were performed between a scale for suicidal risk and the predicted score produced by our model. Based on several novel approaches, we presented a novel graph neural network (GIN)-based model that employs multi-dimensional scale-based prediction of depression and acute SI with a high sensitivity and specificity. This could promote deployment of the model in the real world.

## Results

### Subject clinico-demographics

Across four centers, including university and secondary/tertiary hospitals, 31,720 (mean age 23.64 $\pm$ 3.96 years old; 68.2% male) out of 32,250 participants responded to six self-report questionnaires: the Patient Health Questionnaire-9 (PHQ-9)^25^, Generalized Anxiety Disorder-7 (GAD-7)^26^, State-Trait Anxiety Inventory-State Anxiety (STAI-S, or STAI-X1)^27,28^, the Resilience Appraisal Scale (RAS)^29^, the Rosenberg Self-Esteem Scale (RSES)^30^, and lifetime SA. The total number of positive acute SI cases was 306/31,720 cases (0.965%) across the four different institutions. The rate of acute SI differed between the training/validation and test sets (0.74% vs. 1.24%; *p* < 0.0001). There was a difference in the ages of the individuals in the training/validation and test sets (mean age, 23.17 ± 4.17 vs. 24.23 ± 3.59 years old; *p* < 0.0001) and the gender ratio was different between the training/validation and test sets (79.95% vs. 53.82% male; *p* < 0.0001). For all five scales (i.e., the PHQ-9, GAD-7, STAI-S, RAS, and RSES), the distributions of the scores were different across centers (*p* < 0.0001) as well as between the training/validation and test sets (STAI-S, *p* = 0.01; all others, *p* < 0.0001). The clinico-demographic information is summarized in Supplementary Table 1. In brief (Supplementary Table 1), the incidence of lifetime SI was approximately 10 times higher than the incidence of acute SI. The incidence of lifetime SI, acute SI, and lifetime SAs in the total dataset was 2641 (8.33%), 306 (0.97%), and 437 (1.38%), respectively, out of 31,720 participants. In total, 358 people received structured interviews using the Mini International Neuropsychiatric Interview (MINI)^31^, of which 102 participants were diagnosed with major depressive episodes (MaDEs), accounting for 0.32% of all participants.

### Prediction of MaDEs: external validation

For the test set (Center 4; Seoul National University (SNU)) with true MaDE labels (*n* = 64), the MaDE prediction model achieved a sensitivity, specificity, accuracy, and area under the receiver operating characteristic (ROC) curve (AUC) of 90.91%, 82.76%, 84.06%, and 0.934 (95% confidence interval (CI), 0.874–0.986), respectively. This was done using logistic regression with least absolute shrinkage and selection operator (LASSO) regularization, and the results were 90.90%, 67.24%, 71.01%, and 0.937 (95% CI, 0.881–0.987) when using a support vector machine (SVM). The GIN-MaDE model achieved values of 96.55%, 95.00%, 95.65%, and 0.996 (95% CI, 0.988–1.000), respectively (Table 1).

**Table 1:** Model performance for prediction of major depressive episodes (MaDE) and acute suicidal ideation (SI): model comparison with external validation.

Prediction	Author/Data Source	No. of patients (positive/total, %)	Model	Sensitivity (%)	Specificity (%)	Accuracy (%)	AUC
**MaDE**	Current study	102^a^/31,720 (0.32%)	Logistic regression with LASSO	*90.91*	82.76	84.06	0.934 (0.874–0.986)
			SVM	*90.90*	67.24	71.01	0.937 (0.881–0.987)
			GIN-MaDE	**96.55**	**95.00**	**95.65**	**0.996 (0.988**–**1.000)**
**Acute SI**	Jung et al.^36^	7,443/59,984 (12.4%)	Logistic regression	*78.20*	77.60	77.90	0.851
			SVM	*78.40*	78.90	78.70	0.853
	Current study	306/31,720 (0.97%)	Logistic regression	*49.71*	98.41	97.78	0.740 (0.711–0.771)
			SVM	*15.03*	99.81	98.72	0.574 (0.552–0.597)
			GIN-u1 +	79.77	80.09	80.09	0.868 (0.840–0.892)
			GIN-u1-	72.25	80.74	80.63	0.834 (0.805–0.861)
			GIN-u2 +	55.49	93.84	93.35	0.865 (0.840–0.888)
			GIN-u2-	49.13	**95.51**	**94.91**	0.858 (0.834–0.882)
			GIN-SMOTE +	81.50	73.40	73.50	0.850 (0.826–0.874)
			GIN-SMOTE-	**83.82**	69.08	69.27	0.827 (0.799–0.855)
			Ensemble of GINs + (*the best model*)	76.30	83.35	83.26	**0.878 (0.855**–**0.899)**
			Ensemble of GINs-	69.36	87.12	86.89	0.861 (0.835–0.885)

Bold numbers indicate the best metrics among graph neural network models. Italic numbers indicate lower sensitivity of logistic regression, and SVM models.

^a^A total of 358 people received structured interviews, of which 102 participants were diagnosed with MaDE, accounting for 0.32% of the total 31,720 participants. The results from a state-of-the-art study by Jung et al.^36^ were cited, for which the confidence intervals of AUC were not reported. In the case of GIN for acute SI, metrics were provided from a dataset both including (e.g., GIN-u1 +) and excluding (e.g., GIN-SMOTE-) Item-9 of PHQ-9.

*MaDE* major depressive episode, *SI* suicidal ideation, *AUC* area under the curve, *LASSO* least absolute shrinkage and selection operator, *SVM* support vector machine, *GIN* graph isomorphism network.

### Prediction of acute SI: external validation

Using conventional algorithms as baseline models, the model achieved a sensitivity, specificity, accuracy, and AUC of 49.71%, 98.41%, 97.8%, and 0.740 (95% CI, 0.711–0.771), respectively, when using logistic regression with LASSO regularization and 15.03%, 99.81%, 98.7%, and 0.574 (95% CI, 0.552–0.597) when using an SVM. On the other hand, all the deep learning models including three GIN models and the ensemble of those recorded better performance than the former two models in terms of AUC score; 0.868 (95% CI, 0.840–0.892) for GIN-u1, 0.865 (95% CI, 0.840–0.888) for GIN-u2, 0.850 (95% CI, 0.826–0.874) for GIN-synthetic minority over-sampling technique (SMOTE), and 0.878 (95% CI of 0.855–0.899) for the ensemble model. Furthermore, the best GIN-based model showed an appropriate balance between sensitivity and specificity (76.3% versus 83.4% for the ensemble) (Table 1).

### Attention plots and interpretation

**Figure 2:**
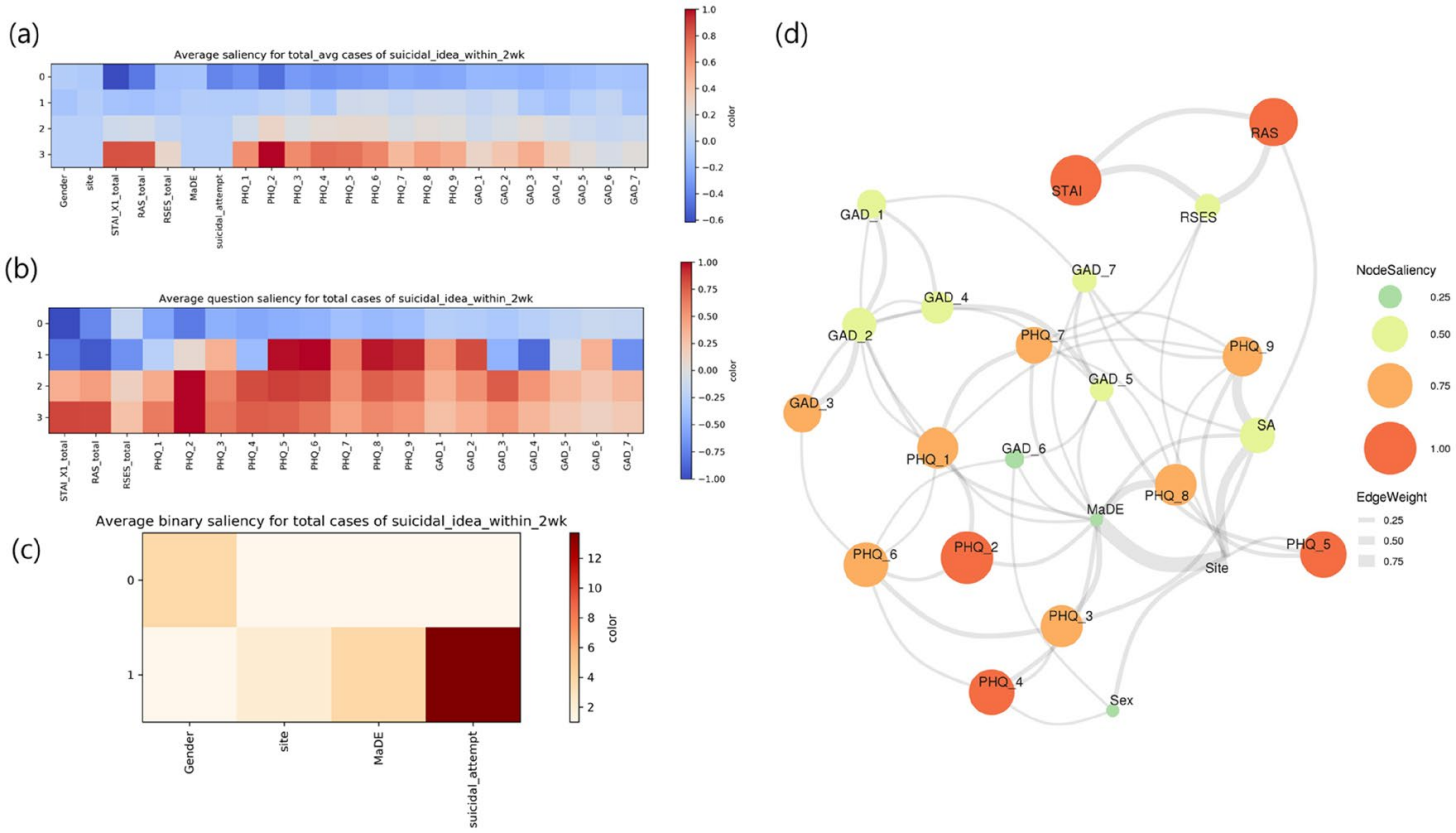
Attention plots: (**a**) Raw averaged attention plots, (**b**) Attention plot comparing the questionnaire items, (**c**) Attention plot comparing the binary items, (**d**) Attention plot for the mixed Gaussian model-based graphical network (generated with the “mgm” R package for visualization) for the questionnaire items on the test set (*n* = 14,238).

The raw averaged attention plots without normalization are given in Fig. 2 for the test set (Fig. 2a). In the attention plot comparing questionnaire items using row-wise normalization (Fig. 2b), a high score (i.e., 4 points) for Item 2 of the PHQ-9 (i.e., PHQ_2, anhedonia) was the most salient positive feature (i.e., a feature that increases the prediction score of acute SI) among the 19 items of the questionnaires. The second most salient positive feature was a high total score for the STAI-S (i.e., the 4th quartile group), which represents a high level of anxiety. For low score (i.e., 1 point, the 1st quartile group), the most salient negative feature (i.e., a feature that decreases the prediction score of acute SI) was a low total score for the STAI-S and a low PHQ_2 score, which means that a low level of anxiety and anhedonia are the most significant feature associated with reduced SI. For intermediate scores (i.e., 2–3 points), the salient positive features were PHQ_5, PHQ_6, and PHQ_8 (i.e., psychomotor symptoms, feeling tired, and trouble concentration on things, respectively) to a similar degree. In PHQ_9, thought that would be better off dead or of hurting, the 1st quartile group decreased SI while the 2nd to 4th showed the opposite result. Point 1 or 2 in RAS total score was the salient negative feature for SI, which means that the 3rd or 4th quartile groups (because of reversed order) of RAS total points decreased SI.

In the attention plot comparing binary items using column-wise normalization (Fig. 2c), the attention values were highest for lifetime SA (odds ratio (OR), 13.69), presence of MaDE (OR, 3.81), female (OR, 3.74), and type of institution (OR, 2.10).

In the plot using the L1-norm of the attention vector, obtained for each column of the 19 questionnaire items (Fig. 2d), the attention values were the highest for PHQ_2 (1.0) and STAI-S had the 2^nd^ highest attention values (0.937). The RAS and PHQ_5 had the 3^rd^ and 4^th^ highest attention values (0.857 and 0.792), respectively. Moreover, the attention plots for the training/validation set showed nearly identical results to those for the test set (Supplementary Fig. 3a–d).

### Ablation study for PHQ-9 item 9

**Figure 3:**
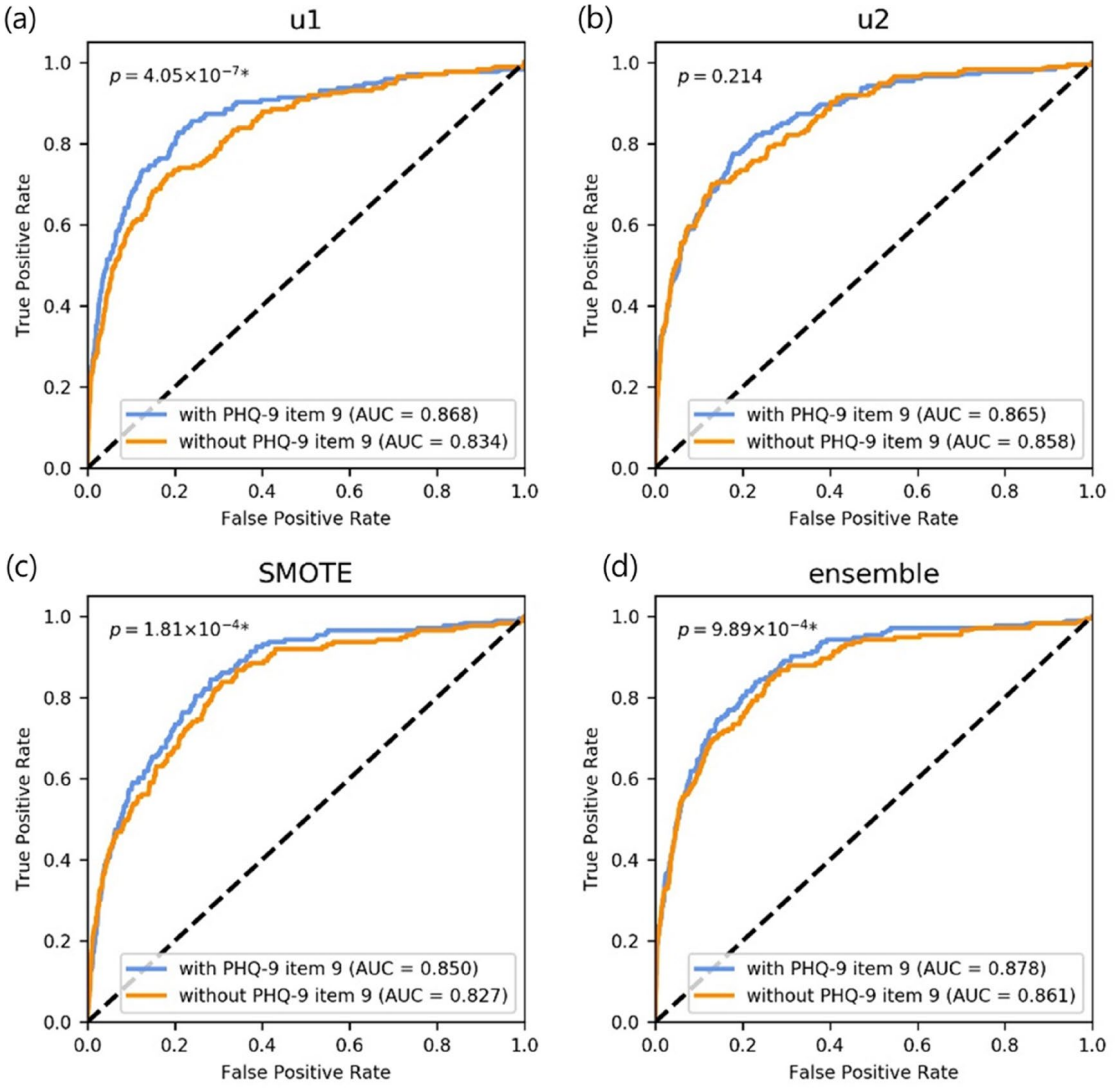
Receiver operating characteristic (ROC) curves of models using (**a**) u1, (**b**) u2, (**c**) SMOTE, and (**d**) ensemble of u1, u2, and SMOTE for ablation study. There were significant but small differences between the diagnostic performance of the model with and without Item 9 of PHQ-9 except for u2. Each *p*-value was calculated from DeLong’s test comparing two ROC curves.

Because PHQ_9 is related to acute SI, the model performance without PHQ_9 was also obtained. In the Ensemble of GIN without PHQ_9, the sensitivity, specificity, accuracy, and AUC were 69.36%, 87.12%, 86.89%, and 0.861 (95% CI, 0.835–0.885), respectively (Table 1). Both accuracy and specificity increased while sensitivity and AUC decreased in the Ensemble of GIN without PHQ_9 (Table 1). There were statistically significant differences between AUCs in models with and without PHQ_9 (AUC = 0.861 vs. 0.878, respectively; *p* < 0.001). The difference was also found in the u1 and SMOTE models (Fig. 3).

### Validity of the labels for acute SI: comparison study

**Figure 4:**
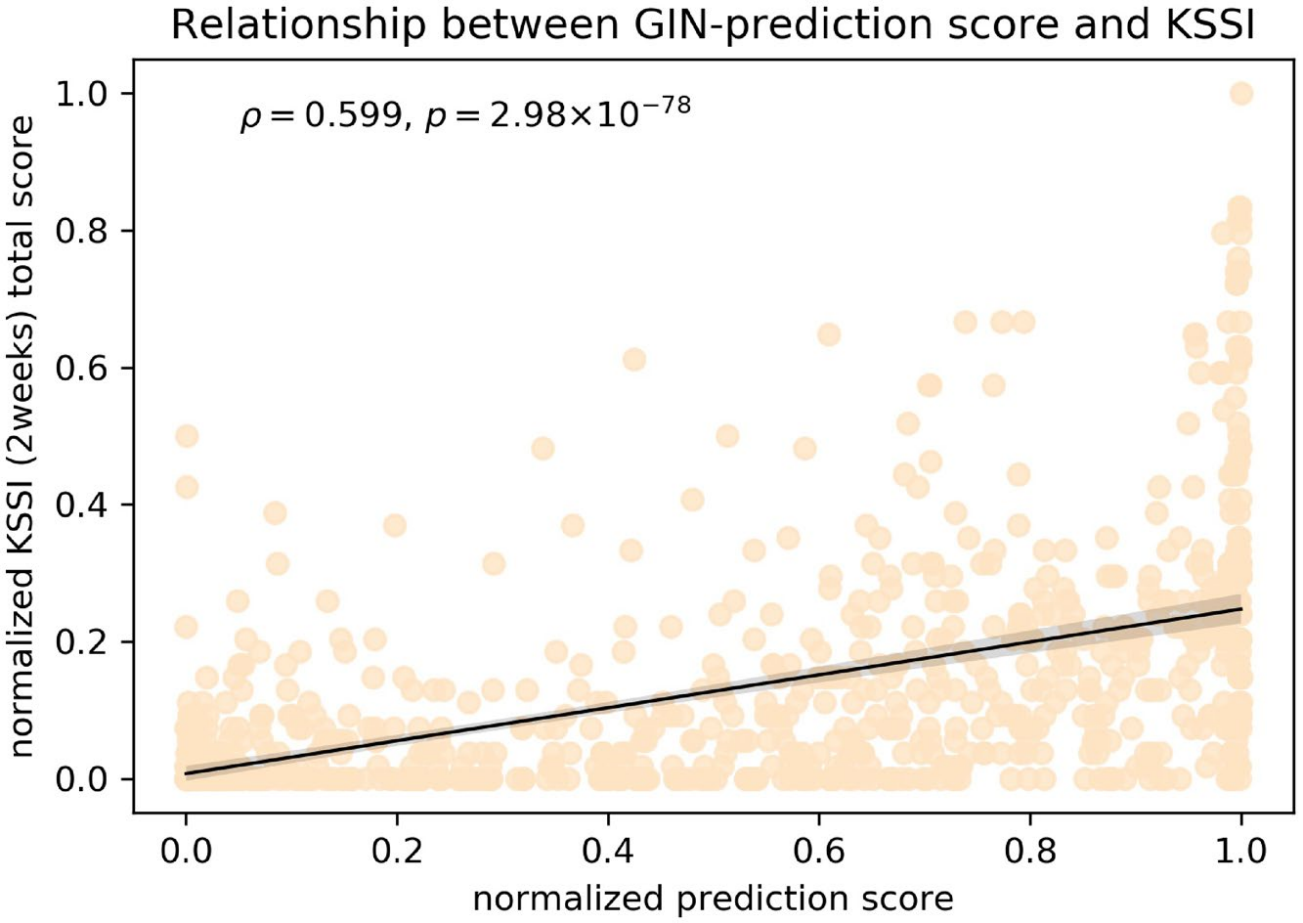
Scatter plot showing the normalized raw prediction score of the model and KSSI total score (*n* = 792 of 13,408; the part of the test set with KSSI scores). Spearman’s correlation coefficient between the prediction score of the model and the KSSI total score was ${\rho }_{\mathrm{pred}}$ = 0.599 (*p* < 0.0001).

Among the subjects in the test set, only *n* = 792 of the 13,408 subjects completed the Korea Advanced Institute of Science and Technology (KAIST) Scale for Suicide Ideation (KSSI). Regarding the Spearman’s rank correlation coefficient 1) between the scores predicted by the best model, the total KSSI score was ${\rho }_{\mathrm{pred}}$= 0.599 (*p* < 0.0001) and 2) between PHQ_9 and the total KSSI score was ${\rho }_{\mathrm{PHQ}}$ = 0.446 (*p* < 0.0001). In the comparison of correlation coefficients,${\rho }_{\mathrm{pred}}$ was larger than ${\rho }_{\mathrm{PHQ}}$ (*p* < 0.0001)^32^. A scatter plot between the raw predicted scores (i.e., the model output *before* applying the sigmoid function) by the best model and the total KSSI score is shown in Fig. 4.

## Discussion

We developed a GNN model to predict acute SI within 2 weeks, which showed improved sensitivity compared to baseline models, and validated it in an external test set: the sensitivity, specificity, accuracy and AUC were 76.3%, 83.4%, 83.3%, and 0.878 (95% CI, 0.855–0.899), respectively, using an ensemble of GIN models with different sampling methods. The values were 15.03%, 99.81%, 98.72%, and 0.574 (95% CI, 0.552–0.597), respectively, using an SVM. Specifically, the best-performing model based on a GIN to predict SI, improved the sensitivity significantly at the cost of reductions in the specificity and accuracy. The low sensitivities of the baseline models prevented the prediction of individuals who might attempt suicide and led to irreversible events^5^. In contrast, the ensemble model achieved a significant increase in sensitivity compared to previous baseline models, allowing more accurate prediction of individuals who might attempt suicide and suggesting that this model could potentially be of great help in the real world.

Our model achieved good performance by incorporating the following three factors. (1) The GNN provides a good graph embedding. GIN^24^ is a variant of a spatial GNN specifically used for graph classification, and extracts an even better representation from the graph than other GNNs, such as graph convolutional networks (GCNs). This is because GINs are equivalent to generalized convolutional neural networks (CNNs) for non-Euclidean data that can be represented as graph structures, such as brain connectivity^33,34^. (2) An ensemble method using under-sampling and over-sampling (i.e., SMOTE for nominal and continuous features (SMOTE-NC)^35^) was designed to handle class imbalance issues. (3) Rich information from multi-dimensional scales and subject clinico-demographic information for large multi-center datasets were used. Seven questionnaires covering domains such as depression, anxiety, resilience, and self-esteem were obtained from *n* = 31,720 individuals across the four centers, which included universities and hospitals. Jung et al.^36^ reported that the baseline models showed good performance in predicting SI over the past 12 months in a young population, with approximately 13 positive cases compared to the current data (12.4% vs. 0.97%, see Table 1). However, it is challenging to predict acute SI within 2 weeks. In the present study, which had severe class imbalance, the SVM without the ensemble method (which is a baseline model) could not extract a good representation of the positive cases, resulting in much lower sensitivity (~ 15%) than the best model*,* while a specificity and accuracy of nearly 99% were achieved. This finding suggests that dealing with class imbalance, such as with the ensemble method, should be considered to prevent prediction bias towards the majority class (i.e., the model always predicts SI-negative). It probably does not matter what kind of model is used, but this analysis is beyond the scope of the current study. Interestingly, our model can show not only feature importance but also the association among features. Although PHQ_2 and STAI-S are features having the highest saliency value, the former was associated with other items of the PHQ-9 and the latter was associated with resilience and self-esteem (Fig. 2d).

We predicted MaDEs as a pseudo-label before the prediction of acute SI because pre-existing psychiatric disorders such as major depressive disorder (MDD) have been known to increase suicide risk^37^. This would be helpful in accurately predicting acute SI. In the MaDE prediction, all the conventional and GIN models achieved AUCs and sensitivities over 90%. This finding suggests that both the PHQ-9 and other scales, including GAD-7, contributed to predicting the MaDE labels. MaDE pseudo-labels were used as input to predict acute SI. Although the presence of a MaDE is 3.81 times more likely to indicate an individual with SI than its absence (Fig. 2c), its low saliency may be indirectly associated with SI via its association with various PHQ-9 items and GAD_7 (“Feeling afraid, as if something awful might happen”) (Fig. 2d and Supplementary Fig. 3d). Interestingly, lifetime SA achieve both high OR among the binary items (Fig. 2c) and a higher saliency score than with MaDE. In addition, MaDE can be accurately predicted with conventional or GIN models. The results suggest that both gathering SA information and predicting MaDE with a model, instead of structural interviews for diagnosis, is an efficient approach for survey-based screening for suicide risk. Moreover, nearly identical attention plots for the training/validation set (Supplementary Fig. 3) and test set (Fig. 2) might suggest that the common “scale and clinico-demographic signature” of acute SI was extracted using the GIN, which models the relationship between the scale items and clinico-demographic information in graph-structured data.

In the attention plots, the model recognized the salient items among the multi-dimensional questionnaires and other information (Fig. 2). Specifically, when comparing 19 questionnaire items, several PHQ-9 items (e.g., items 2, 4, 5, and 6) and the total STAI-S and RAS scores showed high saliency values. Among these features with high saliency, anhedonia (PHQ_2) and high state and trait anxiety (STAI-S total score), were the two most salient features. The PHQ_2 is one of two cardinal symptoms of depression: i.e., PHQ_1 (depressed mood or hopelessness) and PHQ_2 (anhedonia)^38^. Especially, it is known that anhedonia is closely related to current suicidal ideation, even for individuals who do not have psychiatric disorders including depression^39,40^. A high STAI-S total score was also associated with increased acute SI, which is consistent with previous studies showing that both state and trait anxiety increase the suicidal risk^41,42^. Furthermore, in a large population-based longitudinal study, anxiety disorders were found to be independent risk factors for suicidal behaviors (i.e., SI and SA), and an increased risk of SA in combination with a mood disorder was found^2^. It has been reported that resilience protects against symptoms of anxiety and depression, and strongly influences the associations between symptoms and lifestyle factors^43^. This is consistent with the findings that low resilience is strongly associated with mild depression and that psychological resilience is linked to social support^44^, and might lead to increased risk of SI compared to non-depressed subjects. Moreover, low resilience was a risk factor for suicidal behaviors^45^. In our study, a high RAS total score was associated with decreased SI, and vice versa, which is also consistent with a previous study showing that high resilience is one of the most protective features for SAs^29,46^.

In the ablation study of PHQ_9, it was related to acute SI, the best model performance without PHQ_9 showed a statistically significant difference in terms of the AUC compared to the model with this item (AUC = 0.861 vs. 0.878, respectively; *p* < 0.001). While the difference between the mean AUC values was relatively small (0.017), a trade-off was found between sensitivity and specificity. Furthermore, the model with PHQ_9 shows that we may pay attention to the item points from 2^nd^ to 4^th^ quartiles. Thus, we argue that the PHQ_9 is an important input feature without serious degradation of the model’s performance for predicting acute SI. In the validation study of the true labels for acute SI, the model prediction score showed a higher correlation with the KSSI score (i.e., it is a more accurate proxy for acute SI than PHQ_9 is (ρ = 0.599 vs. 0.446), respectively, *p* < 0.001; see the validity of the labels for acute SI section in the Results section). Originally, the PHQ-9 was designed for screening depression and to assess severity, not to assess suicide risk^25^. Interestingly, in a recent validation study, Na et al.^47^ showed that PHQ_9 is an insufficient assessment tool for suicide risk and SI because of the limited utility in certain clinico-demographic and clinical subgroups, which is in line with our results. Our results indicate that our model-based predictions resulting from multi-dimensional information are more valid than those from only a single question (i.e., PHQ_9 and acute SI label) and that those predictions provide an alternative to a structured interview or a scale for suicide risk. While PHQ_9 itself may not be a valid measure for SI, our results (Fig. 2b) suggest that intermediate scores (i.e., 2–3 points) for this item should not be overlooked. This strategy should also apply to PHQ_6 (feeling tired) and PHQ_8 (concentration problem) (Fig. 2b).

It is worth noting that this multi-dimensional scale dataset was collected before the outbreak of COVID-19, and that the specific representation of mental illness, including depression and anxiety, evoked by consequences of the COVID-19 pandemic may not be reflected by the scales used in the present study. Further research is needed to explore the effectiveness of the proposed model during the COVID-19 pandemic. In addition, the true labels for acute SI may be improved if we obtain the labels for suicidal behavior from reference to standards, such as structured interviews by clinicians for all subjects. However, this process is time consuming, impractical, and requires large amounts of research funding.

This study has several limitations. Because prediction of major depressive episodes using small datasets can lead to overfitting, the benefit of the pseudo-label^48^ of MaDE to predict SI should be confirmed in future studies. The significant relationship of predicted scores with KSSI was the result from only a part of test dataset (792 of 13,408), and thus it is likely that the missing data were not randomly missed, and further studies are needed to generalize this. The type of institution cannot be generalized to other types of data obtained from workplaces. Although high saliency of the type of institution is plausible (Fig. 2c), its value for each individual might not be meaningful and must be interpreted carefully. Although beyond the scope of the current study, exploration of the impact of edge and sparsity definitions on performance is necessary. To generalize the results of young adults to other populations, further studies of a wide range of ages are needed. Longitudinal cohort studies with deep graph isomorphism networks that perform better than baseline models are needed to investigate factors that can predict future SAs or new SI cases. Verification studies are needed to determine whether predicting SI instead of SAs is effective in preventing SAs in the real world.

In conclusion, we developed and validated a deep-learning-based compensatory tool by using extracted deep features from multi-dimensional self-report questionnaires covering depression, anxiety, resilience, self-esteem, and clinico-demographic information in a large dataset. This was done to predict suicide risk instantaneously and to monitor responses to suicide prevention strategies. This could be useful in remote clinical practice in the general population of young adults for specific situations such as the COVID-19 pandemic.

## Methods

### Dataset

Young adults between the ages of 18 and 34 years old were included from across the four centers in the Research Consortium for Young Adulthood Depression. The participants underwent medical examinations, including mental health measurements and were enrolled in the study between January 1, 2018, and December 31, 2019. The research protocol for the present study was approved by the Korea Advanced Institute of Science and Technology (KAIST) Institutional Review Boards. The study protocol was performed in accordance with the relevant guidelines. Informed consent was obtained from all the participants. Anxiety disorders are known as independent risk factors for suicidal behavior (i.e., SI and SAs) and increase the risk of SA when combined with mood disorders such as MDD^2^. A history of SAs is considered a crucial predictor of future suicidal behaviour^49,50^. cng the presence of MDD should be very helpful^51^ to improve the diagnostic performance of the prediction model for acute SI, structured interviews given by psychiatrists in large populations are less cost effective. Here, the MINI^31^ was performed on a portion of the participants by four psychiatrists (S.H.K., S.H.Y., D.H.K., and M.S.K.) in centers 1–3 and via a web-based version in Center 4. Then, using data with labels for MaDE, a prediction model (GIN-MaDE) was trained. Finally, the MaDE pseudo-labels were predicted by the trained GIN-MaDE network for participants without MaDE labels (this is detailed in the *Semi-supervised learning-based input features: pseudo-labels for the MaDE* section) and were used to predict acute SI. The presence of acute SI was determined when the participant responded “yes” to the question “Have you ever thought of suicide in the past 2 weeks?” To develop the model, self-report questionnaires and other clinical data from three independent institutions were obtained: Center 1 was KAIST (n = 17,322), Center 2 was Gachon University Hospital (Gachon) (n = 69), and Center 3 was Samsung Medical Center (SMC in Seoul, n = 91). For external validation, questionnaires and other clinical data obtained from Center 4 (Seoul National University, SNU, n = 14,238) were used. All the data were anonymized prior to combining the data from the four institutions. All the descriptions of the self-report questionnaires are detailed in the Supplementary Material. The overall workflow for constructing the graph-structured dataset is illustrated in Fig. 1a.

### GIN as a graph neural network

A GIN is a variant of a GNN with equal representative/discriminative power for graph-structured data, such as the Weisfeiler-Lehman (WL) test. GIN is one of the most powerful existing tests for distinguishing a broad class of graphs^52^; it was developed for graph classification and has achieved state-of-the-art performance^24^. More specifically, for each node, *v*, graph convolution aggregates neighboring node features (or nodes connected by weighted edges), $\sum_{u\in \mathcal{N}(v)}{p}_{u}^{(k-1)}$ (see Eq. 1 and 2 in the Supplementary Material). Then the aggregation is combined with the node feature of the previous hidden layer, ${p}_{v}^{(k-1)}$, to update the node feature at the current *k*-th hidden layer, ${p}_{v}^{(k)}$. Next, for each node, multi-layer perceptron (MLP) layers elevate the node feature to a high-dimensional latent space (i.e., from the dimension of the node features of the hidden layer to the dimension of the MLP layers; ${\mathbb{R}}^{{C}^{\left(k-1\right)}}\to {\mathbb{R}}^{{C}^{\left(k\right)}},\mathrm{ where }\,\,{C}^{\left(k\right)}$ denotes the dimension of the node features of the *k*-th hidden layer). For each hidden graph convolutional layer, all the updated node features were summed to make a graph feature of the *k*-th hidden layer, ${p}_{G}^{\left(k\right)}$, which is known as sum-pooling. For the graph-level readout, all *K* graph features from the hidden layers were concatenated to make a final graph feature, ${p}_{G}$ (Fig. 1), extracting an excellent graph representation^24^ for positive and negative cases of acute SI. Finally, ${p}_{G}$ was fed to the final classifier to calculate the sigmoid prediction score of acute SI. The overall model architecture is illustrated in Fig. 1, and the mathematical equations are described in the Supplementary Material. The time complexity of GIN is $O(m)$ where m is the total number of graph edges. This means that the GIN layer only linearly depends on the number of edges^53^.

### Semi-supervised learning-based input features: pseudo-labels for MaDE

MaDE labels are important information for predicting acute SI; however, only a fraction of MaDE labels were available because only a fraction of subjects, 294 individuals in the training/validation set and 64 individuals in the test set, completed the MINI. Following the pseudo-labeling strategy frequently used in semi-supervised learning^48^, we generated pseudo-labels for MaDE via other questionnaires and clinico-demographic information, (such as gender and type of institution) using the GIN-MaDE network prior to training the GINs for predicting acute SI. Details are described in the Supplementary Methods section.

### Prediction of acute SI: subsampling strategy

To overcome the intrinsic challenge of SI prediction or the sparsity of positive cases of acute SI (i.e., the class imbalance problem), we utilized not only data augmentation for balancing the data but also ensembles of models with different subsamplings. First, a GIN model was developed to predict acute SI using the MaDE pseudo-labels as an additional input feature. Most machine learning models built on imbalanced datasets give predictions that are biased towards the majority class (i.e., negative cases); hence, the model will always predict a case as a negative case even if it is a positive case. Specifically, to obtain different decision boundaries to be ensembled, which may largely depend on the subsampled data distribution, we built three different GIN models with different subsampling strategies: 1) GIN-u1 (under-sampling of the majority class with a balance ratio of 10), 2) GIN-u2 (under-sampling of the majority class with a balance ratio of 5), and 3) GIN-SMOTE^35^ (over-sampling of the minority class with a balance ratio of 1). The majority and minority classes have negative and positive SI labels, respectively, and the balance ratio was defined as the ratio of negative to positive cases in the subsampled data from the training set.

For the training and validation sets, datasets from centers 1–3 (SMC, Gachon, and KAIST) were used, and a dataset from Center 4 (SNU) was used for the test set. Note that the test set was never augmented.

### Ensemble model

After training each of the three GIN models defined above, the best model for each subsampling strategy was saved at the epoch when the model generated minimal validation loss and achieved both validation sensitivity and specificity over 80% to prevent selection of models with too low sensitivity and specificity: GIN-u1-best, GIN-u2-best, and GIN-SMOTE-best. Next, the final ensemble GIN model was obtained using the three best models. Specifically, the sigmoid prediction scores from the best models were averaged to obtain the final prediction score of the ensemble model, which process is known as “soft voting”^54,55^.

### Evaluation

For the prediction of MaDE and acute SI, the sensitivity, specificity, and accuracy were calculated for all the models. To evaluate the diagnostic performance of the models, an ROC analysis was performed to obtain the AUC, and DeLong’s method was used to compare the AUCs. For the comparison with conventional algorithms, logistic regression with LASSO and an SVM (detailed in the Supplementary Material) were used for the prediction of MaDEs and acute SI. All statistical analyses were performed using R version 3.6.1 (R Foundation for Statistical Computing, Vienna, Austria).

### Ablation study for PHQ_9

Because PHQ_9 (“Thoughts that you would be better off dead or of hurting yourself in some way”) is related to acute SI, including PHQ_9 as a predictor could be redundant. Moreover, response to PHQ_9 has been reported to be a moderate predictor of a subsequent SA or death^56^. However, in studies for the validation of PHQ_9 using the Structured Clinical Interview for DSM Disorders (SCID) assessment as the reference standard, it had a good sensitivity, specificity, and negative predictive value. However, it had low positive predictive value (PPV) in irritable bowel disease (20.8%)^57^ and neurological disorders such as epilepsy (39.1%), migraine (54.5%), multiple sclerosis (41.7%), and stroke (57.1%)^58^. Here, to test the benefit of inclusion of PHQ_9, the performance of the model without PHQ_9 was assessed and compared with that of the model including PHQ_9. Specifically, the ROC comparison of the best GIN-based model with and without PHQ_9 was performed using DeLong’s method. The saliency plots were also compared with and without PHQ_9 using the best GIN-based model.

### Validity of the labels for acute SI: comparison study

Self-report instruments for the assessment of suicidal thinking, such as the Beck Scale for Suicidal Ideation, could be a reliable quantitative reference for acute SI^59–61^. The KSSI^10^ is a comprehensive scale to evaluate suicide risk. The KSSI score for the previous 2 weeks was significantly correlated with the Beck Scale for Suicidal Ideation score (Kendall’s τ = 0.35, p < 0.001) in our previous study^10^. Spearman’s correlation coefficients were calculated between the KSSI total score and the prediction score, and also between KSSI total score and PHQ_9. Two correlation coefficients were compared to investigate the reliability of the model prediction score.

### Attention plots and interpretation

To interpret what the ensemble model “thinks” is important for the prediction of acute SI, we calculated the saliency/attention values. These are defined as the gradient of the input with respect to the model output, $\frac{\partial y}{\partial {x}_{i}}$, where *y* is the linear output of the prediction model and ${x}_{i}$ is the *i*-th input node feature for $\mathrm{i}=\mathrm{0,1}, \ldots , N$ (*N* = the number of nodes in the graph). This shows how much the output changes when we change the input values. The set of attention plots was obtained for both the test set (Fig. 2) and the training/validation set (Supplementary Fig. 3).

## Supplementary Information

Supplementary Information.

## Data Availability

Due to potentially identifying information, the data that support the findings of this study are not publicly available, but can be obtained under the conditions of reasonable request to corresponding authors and the permission of the Institutional Review Board.

## Code availability

The codes are available at https://github.com/kyuchoi/graph_neural_network_suicide_prediction.
