# Author Correction: Deep graph neural network-based prediction of acute suicidal ideation in young adults

**DOI:** 10.1038/s41598-021-99825-5

**Published:** 2021-10-06

**Authors:** Kyu Sung Choi, Sunghwan Kim, Byung‑Hoon Kim, Hong Jin Jeon, Jong‑Hoon Kim, Joon Hwan Jang, Bumseok Jeong

**Affiliations:** 1 Graduate School of Medical Science and Engineering, Korea Advanced Institute for Science and Technology (KAIST), 291 Daehak‑ro, Yuseong‑gu, Daejeon, 34141 Republic of Korea; 2 Department of Psychiatry, Yonsei University College of Medicine, Seoul, Republic of Korea; 3 Department of Bio and Brain Engineering, Korea Advanced Institute for Science and Technology (KAIST), Daejeon, Republic of Korea; 4 Department of Psychiatry, Depression Center, Samsung Medical Center, Sungkyunkwan University School of Medicine, Seoul, Republic of Korea; 5 Department of Psychiatry, Gil Medical Center, Gachon University College of Medicine, Gachon University, Incheon, Republic of Korea; 6 Neuroscience Research Institute, Gachon Advanced Institute for Health Science and Technology, Gachon University, Incheon, Republic of Korea; 7 Department of Human Systems Medicine, Seoul National University College of Medicine, 103 Daehak‑ro, Jongro‑gu, Seoul, 03080 Republic of Korea; 8 KAIST Institute for Health Science and Technology, Korea Advanced Institute for Science and Technology (KAIST), Daejeon, Republic of Korea; 9 KAIST Clinic Pappalardo Center, Korea Advanced Institute for Science and Technology (KAIST), Daejeon, Republic of Korea

Correction to: *Scientific Reports* https://doi.org/10.1038/s41598-021-95102-7, published online 04 August 2021

The Code availability section in the original version of this Article was incomplete.

“Codes will be uploaded soon to [Insert URL].”

now reads:

“The codes are available at https://github.com/kyuchoi/graph_neural_network_suicide_prediction”

The original Article has been corrected.

