# Supplementary material for: Deep graph neural network-based prediction of acute suicidal ideation in young adults
Source: Sci Rep. 2021 Aug 4;11:15828. doi: 10.1038/s41598-021-95102-7 (PMC8338980; doi:10.1038/s41598-021-95102-7)
Supplement: Supplementary file 1 — Supplementary Information. [file 41598_2021_95102_MOESM1_ESM.docx]

**Title: Deep graph neural network-based prediction of acute suicidal ideation in young adults**

Kyu Sung Choi, M.D.^1^, Sunghwan Kim, M.D.^1^, Byung-Hoon Kim, M.D.^2,3^, Hong Jin Jeon, M.D., Ph.D.^4^, Jong-Hoon Kim, M.D., Ph.D.^5,6^, *Joon Hwan Jang, M.D., Ph.D.^7^, *Bumseok Jeong, M.D., Ph.D.^1,8,9^

1. Graduate School of Medical Science and Engineering, Korea Advanced Institute for Science and Technology (KAIST), Daejeon, Republic of Korea
2. Department of Psychiatry, Yonsei University College of Medicine, Seoul, Republic of Korea
3. Bio Imaging and Signal Processing Laboratory, Department of Bio and Brain Engineering, Korea Advanced Institute for Science and Technology (KAIST), Daejeon, Republic of Korea
4. Department of Psychiatry, Depression Center, Samsung Medical Center, Sungkyunkwan University School of Medicine, Seoul, Republic of Korea
5. Department of Psychiatry, Gachon University College of Medicine, Gil Medical Center, Gachon University, Incheon, Republic of Korea
6. Neuroscience Research Institute, Gachon Advanced Institute for Health Science and Technology, Gachon University, Incheon, Republic of Korea
7. Department of Human Systems Medicine, Seoul National University College of Medicine, Seoul, Republic of Korea
8. KAIST Institute for Health Science and Technology, Korea Advanced Institute for Science and Technology (KAIST), Daejeon, Republic of Korea
9. KAIST Clinic Pappalardo Center, Korea Advanced Institute for Science and Technology (KAIST), Daejeon, Republic of Korea

**Corresponding Author:**

*Bumseok Jeong, M.D., Ph.D.

Graduate School of Medical Science and Engineering, Korea Advanced Institute for Science and Technology,

291 Daehak-ro, Yuseong-gu, Daejeon, 34141, Republic of Korea

Tel: 82-42-350-4245

E-mail: bs.jeong@kaist.ac.kr

*Joon Hwan Jang, M.D., Ph.D.

Department of Human Systems Medicine, Seoul National University College of Medicine, 103 Daehak-ro, Jongro-gu, Seoul, 03080, Republic of Korea

Tel: 82-2-880-9022

E-mail: jhjang602@snu.ac.kr

**Supplementary Material**

**Methods and materials**

*Baseline algorithms for prediction models: logistic regression with LASSO regularization and SVM*

As baseline algorithms for prediction models*,* logistic regression with least absolute shrinkage and selection operator (LASSO) and a support vector machine (SVM) were selected for comparison with our graph isomorphism network (GIN)-based ensemble model^1^.

For classic binary classification, logistic regression is the most widely used, simple linear model. LASSO is also a linear model that estimates sparse coefficients by reducing the size of the coefficients and selecting the remaining non-zero terms after regularization. In other words, LASSO effectively “selects” the important features by minimizing the residual sum of squares, subject to the sum of the absolute values of the coefficients being less than a constant^2^. In addition, the SVM maps input vectors into certain high-dimensional feature spaces through a non-linear mapping scheme, such as Gaussian and polynomial mapping^3^, for binary classification. In the feature space, a linear decision boundary is constructed that is applicable to both separable and non-separable training data and takes advantage of the property of the decision space to guarantee high generalizability during machine learning^3^.

*Self-report questionnaires on depression, anxiety, and clinical characteristics: PHQ-9, GAD-7, STAI-S, RAS, RSES, and KSSI*

To provide sufficient information about the mental states of the subjects, five scales (i.e., the PHQ-9^4^, GAD-7^5^, STAI-S^6,7^, RAS^8^, and RSES^9^) and two labels (lifetime SA and the true or pseudo-label for MaDE) were each involved in assessing multi-dimensional aspects of SI, including depression, symptoms associated with GAD, status and trait anxiety, resilience, self-esteem, MaDEs, and suicide risk. More specifically, 23 input features were used to train and test prediction models for acute SI: gender and type of institution comprised 2 input features; individual item of PHQ-9 and GAD-7 comprised 16 input features; total scores for STAI-S, RAS, and RSES comprised 3 input features (i.e., STAI-S_total, RAS_total, and RSES_total); the MaDE label comprised 1 input feature; and the lifetime SA label comprised 1 input feature. Each item of a scale is referred to as (scale name)_(item number), i.e., PHQ_1, PHQ_2 …, and PHQ_9 indicate the Item 1, 2, …, and 9 of the PHQ-9, respectively. All the input features were one-hot encoded as four-dimensional vectors because the number of possible answers to each item of the PHQ-9 and GAD-7 was four (i.e., 0-3 points)^4,5^. Gender was defined as binary: 0 if the subject was male and 1 if the subject was female. The type of institution was defined as binary: 0 if the institution is in a university setting, which has a lower incidence of depression, or 1 if the institution is in a hospital setting, which has a higher incidence of depression. The type of institution was employed as an input feature to provide information on different incidences of depression or the distribution of data to the model.

*PHQ-9*

The Patient Health Questionnaire-9 (PHQ-9)^4^ is a nine-item instrument that screens for the presence and severity of depression. It asks patients about their experiences over the preceding 2 weeks. Scores range from 0 to 27. In general, a score ≥10 suggests depression. The PHQ-9 is an instrument for making criteria-based diagnoses of depressive and other mental disorders commonly encountered in primary care, based on 9 items upon which the diagnosis of depressive disorders in the Diagnostic and Statistical Manual of Mental Disorders, 4th Edition (DSM-IV), is based^4^. Major depressive disorder (MDD) is diagnosed if 5 or more of the 9 depressive symptom criteria have been present for at least “more than half the days” in the past 2 weeks. As a severity measure, the score can range from 0 to 27 because the scores for each item range from 0 (not at all) to 3 (nearly every day). The higher the total score is, the higher severity of depressive mood. The Korean version of the PHQ-9 is a reliable and valid tool for the screening and assessment of depressive patients, showing a high test-retest correlation coefficient (r=0.89, p<0.01)^10^.

*STAI-S*

The State-Trait Anxiety Inventory-State Anxiety (STAI-S)^6,7^ is a psychological inventory that measures state anxiety (i.e., anxiety about an event) and consists of 20 questions. The scores range from 20 to 80, and higher scores mean higher levels of anxiety. More specifically, the STAI-S evaluates state anxiety (S-Anxiety), which is the transient anxious state of a subject at the time of the experiment and may be influenced by external factors, while trait anxiety (T-Anxiety) reflects generally experienced anxiety and shows an individual’s characteristic response to anxiety. Responses to individual items were measured on a 4-point Likert scale from 1 (not at all) to 4 (very much so), with the total score ranging from 20 to 80. The higher the total score was, the higher the level of state and trait anxiety. The Korean version of the STAI has been validated and found to have appropriate psychometric properties and good internal consistency, showing Cronbach’s α = 0.92 for the SAI and 0.90 for the TAI^6^.

*GAD-7*

The Generalized Anxiety Disorder-7 (GAD-7)^11^ is a self-report instrument used to assess the severity of anxiety in general. The GAD-7 has seven items, and the scores range from 0 to 21. Higher scores indicate higher anxiety over the preceding 2 weeks. As a 7-item anxiety scale for generalized anxiety disorder (GAD), the GAD-7 is not only a strong means of identifying cases of GAD but also an excellent severity measure, with higher scores on the GAD-7 are strongly associated with multiple domains of functional impairment and disability days (Cronbach’s α for internal consistency = 0.92)^5^. Although GAD and depressive symptoms commonly co-occur, a factor analysis revealed them as distinct dimensions, which ensures that the GAD-7 has independent input features different from PHQ-9, allowing it to provide our best model with more information about a subject’s mental state. Each item is scored from 0 (not at all) to 3 (nearly every day), with a total score ranging from 0 to 21. The Korean version of the GAD-7 also showed acceptable diagnostic accuracy and internal consistency (Cronbach’s α = 0.93)^11^.

*RAS*

The Resilience Appraisal Scale (RAS)^8^ is used to assess an individual's ability to cope with his or her emotions, solve problems, and acquire social support. It consists of 12 items, and the scores range from 12 to 60. Higher scores indicate more positive self-appraisal. An individual’s ability to cope with emotions, solve problems, and gain social support are known as 3 types of positive self-appraisal that are particularly important in buffering individuals from suicidal ideation (SI) when facing stressful events. The RAS was developed to measure these appraisals quatitatively^8^. Reflecting the three areas, 12 items were developed and are scored from 1 (strongly disagree) to 5 (strongly agree). The alpha value of reliability was 0.88 for the scale. The Korean version of the RAS has been validated, showing a mean score of the general population was signiﬁcantly higher than that of psychiatric outpatients and an RAS has high internal consistency (Cronbach’s α = 0.92)^12^.

*RSES*

The Rosenberg Self-Esteem Scale (RSES)^9^ consists of 10 items answered using a four-point scale to measure feelings of worthiness. The scores range from 10 to 40, and higher scores indicate higher self-esteem. Self-esteem represents a positive or negative attitude towards oneself. Rosenberg et al.^9^ revealed that people who have high self-esteem feel more happiness, are more stable, and have goal-driven tendencies, whereas those who have low self-esteem become angry more frequently, feel unhappiness, and are annoyed more frequently. The RSES^9^ evaluates self-esteem on 10 items (i.e., 5 items expressed are positive statements, and 5 items expressed are negative statements), scored from 0 (strongly disagree) to 4 (strongly agree). The Korean version of the RSES was validated and has a high internal consistency (Cronbach’s α = 0.90)^13^.

*MINI*

The Mini International Neuropsychiatric Interview (MINI) is a widely used, brief structured diagnostic interview for major psychiatric disorders in the Diagnostic and Statistical Manual of Mental Disorders (DSM). It was validated for reliability by comparing the MINI to the Structured Clinical Interview for DSM-III-R-Patient Version. The MINI showed similar performance in a shorter period of time^14^.

*KSSI*

The KAIST Scale for Suicidal Ideation (KSSI) was developed and validated on college students via mental health screening questionnaires^15^. Shim et al. reported that several factors, including low self-esteem, may enhance the prediction of future SI^15^. The KSSI comprises 28 items: 14 items on the past 2 weeks and another 14 items on the past 1 year. In the current study, the KSSI total score was defined as the sum of the scores on the first 14 items, rated from 0 to 4 to assess various levels of SI over the past 2 weeks on a scale ranging from mild (“I would rather fall asleep and not wake up”) to severe (“I will carry out my thoughts of wanting to take my own life”).

*Graph construction*

A graph is defined as $\mathcal{G}=\left( \mathcal{V,E} \right)$, where $\mathcal{V}=\left\{ v_{1},v_{2}, \ldots, v_{N} \right\}$ is a set of vertices, or nodes, and $\mathcal{E=[}e_{ij}]$ is a set of edge weights, where edge $e_{ij}$ connects vertices $v_{i}$ and $v_{j}$, if they are adjacent ($i, j=1,2, \cdots, N)$. $X=\{x_{1},x_{2}, \ldots, x_{N}\}\in\mathbb{R}^{N\times D}$ is a set of node feature vectors $x_{i}$ for $v_{i}\mathcal{\in V}$, where *D* is the dimension of the node feature vector. A = ${[e}_{ij}]\in\mathbb{R}^{N\times N}$is an adjacent matrix with edge weights as components. More specifically, $e_{ij}=\frac{COV(r_{x_{i}},r_{x_{j}})}{{r_{\sigma_{x_{i}}}}{r_{\sigma_{x_{j}}}}}$ is the covariance of rank variables when the node feature vectors are $r_{x_{i}} \mathrm{and} r_{x_{j}}$, and the standard deviations of the rank variables of the node feature vectors are ${r_{\sigma_{x_{i}}}}\mathrm{and} {r_{\sigma_{x_{j}}}}$, or Spearman’s rank correlation coefficient when the node feature vectors are ordinal variables (i.e., gender, type of institution, lifetime SA, PHQ_1, PHQ_2 …, PHQ_9, GAD_1, GAD_2 …, GAD_7). Similarly, Pearson’s correlation coefficient, or $e_{ij}=\frac{COV(x_{i}, x_{j})}{{\sigma_{x_{i}}}{\sigma_{x_{j}}}}$, was used for edge weights when the node feature vectors were continuous variables, i.e., for the STAI-S_total, RAS_total, and RSES_total scores. For the prediction of MaDEs and acute SI, seven questionnaires obtained from individual subjects were used to construct a graph, $\mathcal{G}$. Because each item was an ordinal variable ranging from 0-3 for two questionnaires, the PHQ-9 and GAD-7, the node feature vectors of the graph, $x_{i}$, is a one-hot encoded vector of the point of an individual item (i.e., 0-3 points). The item values of the STAI-S, RAS, and RSES questionnaires were summed and discretized into values from 0-3 using quantiles to make the total scores ordinal variables.

For the RAS and RSES, the ordinality was flipped (i.e., higher total scores were discretized to 1, and lower total scores were discretized to 4) to make all the correlation coefficients or edge weights non-negative, considering that the RAS and RSES were negatively correlated with acute SI, which was investigated via exploratory data analysis (Supplementary Fig. 1).

The edge weights between the nodes, $a_{ij}$, were calculated from Spearman’s rank correlation coefficients between the node features before one-hot encoding. More specifically, the edges were calculated from the pairwise correlation coefficients between individual item values (i.e., 0-3 points) of the questionnaire using the *qgraph* R package for the training and validation set. To prevent edges in the graph from being non-negative, the absolute values of the edge weights were used. The sparsity of the graph edges was controlled by setting the threshold to 0.6; i.e., edges with correlation coefficients < 0.6 were removed.

Finally, questionnaires obtained from individual subjects were used to construct a graph, in which the number of nodes was 23, and the number of edges was 127 with non-zero, positive edge weights for each graph. For validation and testing, the same edge matrix obtained from the training set was used for graph construction in the validation and test sets.

*Mathematical process of GNN*

More specifically, a GNN aggregates neighboring node features and combines the aggregation with the previous node feature, namely, aggregate and combine functions. The GIN specifies the function for aggregation and combination as an addition operation, and multi-layer perceptron (MLP) layers are added for high-dimensional transformation^16^. More specifically, K hidden graph convolutional layers were used (Eq. 2): the aggregate and combine functions from Eq. 1 were replaced by the addition operation and combined with an MLP.

$a_{v}^{\left( k \right)}=\mathrm{AGGREGAT}E^{\left( k \right)}\left( \left\{ h_{u}^{\left( k-1 \right)}:u\in\mathcal{N}\left( v \right) \right\} \right), h_{v}^{\left( k \right)}=\mathrm{COMBINE}^{\left( k \right)}\left( h_{v}^{\left( k-1 \right)}, a_{v}^{\left( k \right)} \right)$ (Eq. 1)

$h_{v}^{(k)}=MLP^{\left( k \right)}(\left( 1+\epsilon^{\left( k \right)} \right)\cdot h_{v}^{\left( k \right)}+\sum_{u\in\mathcal{N}\left( v \right)} h_{u}^{\left( k-1 \right)})$ for $k=0,1, \cdots, K$(Eq. 2)

where $h_{v}^{\left( k \right)}$ is the feature vector of node $v\mathcal{\in V}$ in graph $\mathcal{G}=\left( \mathcal{V,E} \right)$ at the *k*th hidden layer; $\mathcal{N}(v)$ indicates neighboring nodes of node $v$, which are decided by the edge features, $\mathcal{E}$; and $\epsilon^{\left( k \right)}$ is a learnable parameter at the *k*th hidden layer. For graph classification, the READOUT function aggregates node features from the final layer to obtain the representation of the entire graph, $h_{G}$:

$h_{G}=\mathrm{CONCAT}\left( \mathrm{READOUT}\left( \left\{ h_{v}^{\left( k \right)} | v\in G \right\} \right) | k=0,1,\cdots,K \right)$ (Eq. 3)

In the GIN, READOUT is replaced by sum-pooling of all the node features from the same layers and concatenation of the sum-pooled features across all the layers to obtain $h_{G}$. Finally, $h_{G}$ is fed into the final classifier to obtain the sigmoid prediction score for acute SI.

*Pseudo-labels for MaDE*

For the GIN-MaDE networks, items of the PHQ-9 (9 items), GAD-7 (7 items), and STAI-S (20 items) were one-hot encoded as 4-dimensional vectors (0-3 points) to create 36 input node features in total; the 36 nodes were constructed into a graph and the correlation coefficients between ordinal variables of nodes (i.e., PHQ_1 and GAD_1 given as 0-3 points) were calculated in the same way as described in the *Graph construction* section to construct the edge features. The labels were binary (0 or 1) according to whether the MaDE was negative or positive; there were 79 positive cases out of a total of *n*=294. The data from center 4, which were used for the external validation of the GIN SI model, were used as the test set; there were 23 positive cases out of *n*=64. The data from the remaining three centers (1-3) were used as the training and validation set; the positive cases in the training set were augmented approximately 3 times using the synthetic minority over-sampling technique (SMOTE)^17^ (detailed in the following section), and randomly divided at an 8:2 ratio. After training, MaDE pseudo-labels were generated for all subjects without true MaDE labels by forward-passing their PHQ-9, GAD-7, and STAI-S items as input node features into the trained GIN-MaDE network.

*Detailed subsampling strategy*

For under-sampling, random subsampling of the majority class was used. For over-sampling, we augmented the training data with positive labels (i.e., subjects with SI) using a strategy called SMOTE^17^. These subsampling strategies were used to address class imbalance in the dataset – the ratio of positive to negative cases was nearly 1:100 – because of the low incidence of acute SI. In the binary classification task, the larger the difference between the number of samples of the majority and minority classes is, the worse the model performs. Additionally, the more data we have, the better the model performs. Specifically, SMOTE introduces synthetic examples by taking the *k* nearest neighbor minority samples and selecting a random point along the line segment between them or taking a linear combination of neighbors in the “feature space” rather than the “data space”^17^. Because our dataset contains both ordinal and continuous variables, we used SMOTE for nominal and continuous features (SMOTE-NC). In SMOTE-NC, the categories of a new synthetic sample are selected as the most frequent category of the nearest neighbors present during generation^18^. In this way, we can augment the training data and balance the number in each class.

*Implementation details*

For the GIN models, 5 graph convolutional layers, combined with the MLP of two 128-dimensional hidden linear layers, were used; i.e., K=5 in Eq. 2. A one-dimensional batch normalization layer and rectified linear units (ReLU) function were applied as the activation function at the end of each hidden layer. Dropout was applied to the final hidden layer to prevent overfitting (rate = 0.5)^19^. A sigmoid function was applied to the final prediction score to achieve binary classification. The binary cross-entropy function was used as an objective function. The adaptive moment estimation (Adam)^20^ optimizer was used with an initial learning rate of 0.01 and decayed by gamma = 0.2 every 10 epochs. The minibatch size was 64, and the models were trained for 50 epochs; the best model was saved using early stopping. The model was implemented using Python 3.6.0, PyTorch 1.4.0, and Deep Graph Library (DGL) 0.4.0^21^ and trained on a workstation equipped with a Tesla P-100 GPU (NVIDIA, Champaign, IL).

*T-SNE*

To visualize the high-dimensional latent feature space in the two-dimensional plane, t-distributed stochastic neighbor embedding (t-SNE) was used^22^. T-SNE maps the pairwise similarity between data points in high-dimensional space and is capable of capturing the local structures as well as revealing the global structures such as the presence of clusters^22^. Because the last layer of a GIN model contains the most encoded latent features, a t-SNE plot was obtained using the high-dimensional features in the last layer just before the last fully connected layer to output the sigmoid prediction score. For each of the three GIN models and the ensemble GIN model, t-SNE plots were obtained for both the validation and test sets.

**Results**

*Attention plots in training/validation set*

A two-dimensional matrix of attention values, or an attention plot, was obtained for each subject and averaged across all the subjects in specific datasets (i.e., the training/validation set or test set). For better visualization of the results, the averaged attention plot was converted using a different normalization method. The following set of attention plots was constructed: 1) raw averaged attention plot without normalization (Fig. 2a); 2) attention plot comparing questionnaire items using row-wise normalization or dividing the raw averaged attention plot by the largest absolute values of each row (only for 19 questionnaire items, Fig. 2b); 3) attention plot comparing binary items by using column-wise normalization or dividing the raw averaged attention plot by the smallest absolute values of each column for only 4 binary categorical clinico-demographic variables (i.e., gender, type of institution, MaDE, and lifetime SA; Fig. 2c); and 4) bar plot obtained using the L1-norm of the attention vector, which is obtained for each column of the 19 questionnaire items and 4 clinico-demographic variables (Fig. 2d).

The overall findings in the training/validation set were similar to those in the test set. The raw averaged attention plots without normalization are given in Supplementary Fig. 3a for the training/validation set. In the attention plot comparing questionnaire items using row-wise normalization (Supplementary Fig. 3b), a high score (i.e., 4 points) for PHQ_2 was the most salient positive feature, and a high total score for the STAI-S (i.e., the 4^th^ quartile group) was the 2^nd^ most salient positive feature among 19 items of the questionnaires. In the attention plot comparing binary items using column-wise normalization, the attention values were highest for lifetime SA (OR, 31.8), presence of MaDE (OR, 7.88), and type of institution (OR, 1.24); OR for female sex was 2.98 (Supplementary Fig. 3c). The L1-norm of the attention vector was obtained for each column of the 19 questionnaire items (Supplementary Fig. 2d).

*T-SNE*

In the validation and test sets, subjects with acute SI (i.e., positive cases) were well classified by all the GIN models (Supplementary Fig. 4). In the validation set, positive cases were well clustered in the lower half, right lower half, and left lower space (Supplementary Figs. 4b-d), and the numbers of positive and negative cases in the training/validation set were 129 and 1290, 129 and 645, and 17,353 and 17,353, respectively, which were controlled by the balance ratio (Supplementary Table 1) for the GIN-synthetic minority over-sampling technique (SMOTE), GIN-u1, and GIN-u2 models, respectively. In the test set using the ensemble GIN model, positive cases were well clustered to the right of center (Supplementary Fig. 4a), although the number of positive cases was too small to recognize compared to the validation set. In the test set, there were 177 positive and 14,061 negative cases (positive ratio, 1.24%) (Supplementary Table 1)

*Elapsed time for training*

The time spent on training was the highest for SMOTE (about 614 seconds per epoch on average), followed by u1 (97 seconds per epoch on average) and u2 (20 seconds per epoch on average). This difference in training time is due to the use of synthetic (SMOTE) or reduced (u1 and u2) numbers of training samples. There were no significant differences in the two cases when comparing with and without item 9 of the PHQ-9 scale (p>0.23; via two sample independent t-test). These results are displayed in Supplementary Fig. 2.

**Supplementary Table 1.** Demographics of subjects

| **Dataset** | **Train/valid** | | | | **Test** | **Total** | ***p*-value** |
| --- | --- | --- | --- | --- | --- | --- | --- |
| **Center/**  **item** | **Center 1** (*n*=17,322) | **Center 2** (*n*=69) | **Center 3** (*n*=91) | **Centers 1+2+3**  (*n*=17,482) | **Center 4**  (*n*=14,238) | **Centers 1+2+3+4**  (*n*=31,720) | **Train/valid vs**  **Test** |
| Age (years)^*^ | 23.15$\pm$4.17  (16-51) | 24.65$\pm$3.81  (19-37) | 25.96$\pm$3.89  (19-35) | 23.17$\pm$4.17  (16-51) | 24.23$\pm$3.59  (18-35) | 23.64$\pm$3.96  (16-51) | <0.0001 |
| Gender  (Male)^**^ | 13,888  (80.18%) | 31  (44.93%) | 57  (62.64%) | 13,976  (79.95%) | 7663 (53.82%) | 21,639 (68.22%) | <0.0001 |
| Suicidal ideation (lifetime)^**^ | 1029  (5.94%) | 30  (43.48%) | 25  (27.47%) | 1,084  (6.20%) | 1,557  (10.94%) | 2,641 (8.33%) | <0.0001 |
| Acute suicidal ideation^**^ | 107  (0.62%) | 11  (15.94%) | 11  (12.09%) | 129  (0.74%) | 177  (1.24%) | 306  (0.97%) | <0.0001 |
| Suicidal attempt  (lifetime)^**^ | 127  (0.73%) | 14  (20.29%) | 9  (9.89%) | 150  (0.86%) | 287  (2.02%) | 437  (1.38%) | <0.0001 |
| MaDE | 8  (0.046%) | 29  (42.03%) | 42  (46.15%) | 79  (0.45%) | 23  (0.16%) | 102  (0.32%) | <0.0001 |
| PHQ-9^*^ | 1.65$\pm$2.89  (0-27) | 8.42$\pm$8.20  (0-22) | 7.09$\pm$6.99  (0-25) | 1.71$\pm$3.02  (0-27) | 2.90$\pm$3.77  (0-27) | 2.23$\pm$3.42  (0-27) | <0.0001 |
| GAD-7^*^ | 1.04$\pm$2.33  (0-21) | 5.00$\pm$6.21  (0-21) | 4.81$\pm$5.55  (0-21) | 1.08$\pm$2.41  (0-21) | 1.96$\pm$3.12  (0-21) | 1.46$\pm$2.77  (0-21) | <0.0001 |
| STAI-S^*^ | 38.16$\pm$10.03  (20-80) | 38.12$\pm$7.14  (23-53) | 49.21$\pm$9.65  (20-76) | 38.22$\pm$10.06  (20-80) | 38.46$\pm6$.50  (20-80) | 38.23$\pm$8.69  (20-80) | 0.01 |
| RAS^*^ | 48.29$\pm$8.46  (0-60) | 40.41$\pm$10.87  (18-60) | 41.03$\pm$11.02  (12-60) | 48.22$\pm$8.52  (0-60) | 46.01$\pm$7.98  (0-60) | 47.26$\pm$8.36  (0-60) | <0.0001 |
| RSES^*^ | 31.95$\pm$5.24  (0-40) | 24.45$\pm$2.79  (17-29) | 27.53$\pm$7.35  (12-40) | 31.90$\pm$5.28  (0-40) | 25.04$\pm$2.47  (0-40) | 28.91$\pm$5.47  (0-40) | <0.0001 |

* indicates mean $\pm$ standard deviation (range)

** indicates positive (or male)/total subjects of the group, and numbers in parenthesis indicates percentage (%).

*Abbreviations*: MaDE, Major depressive episode


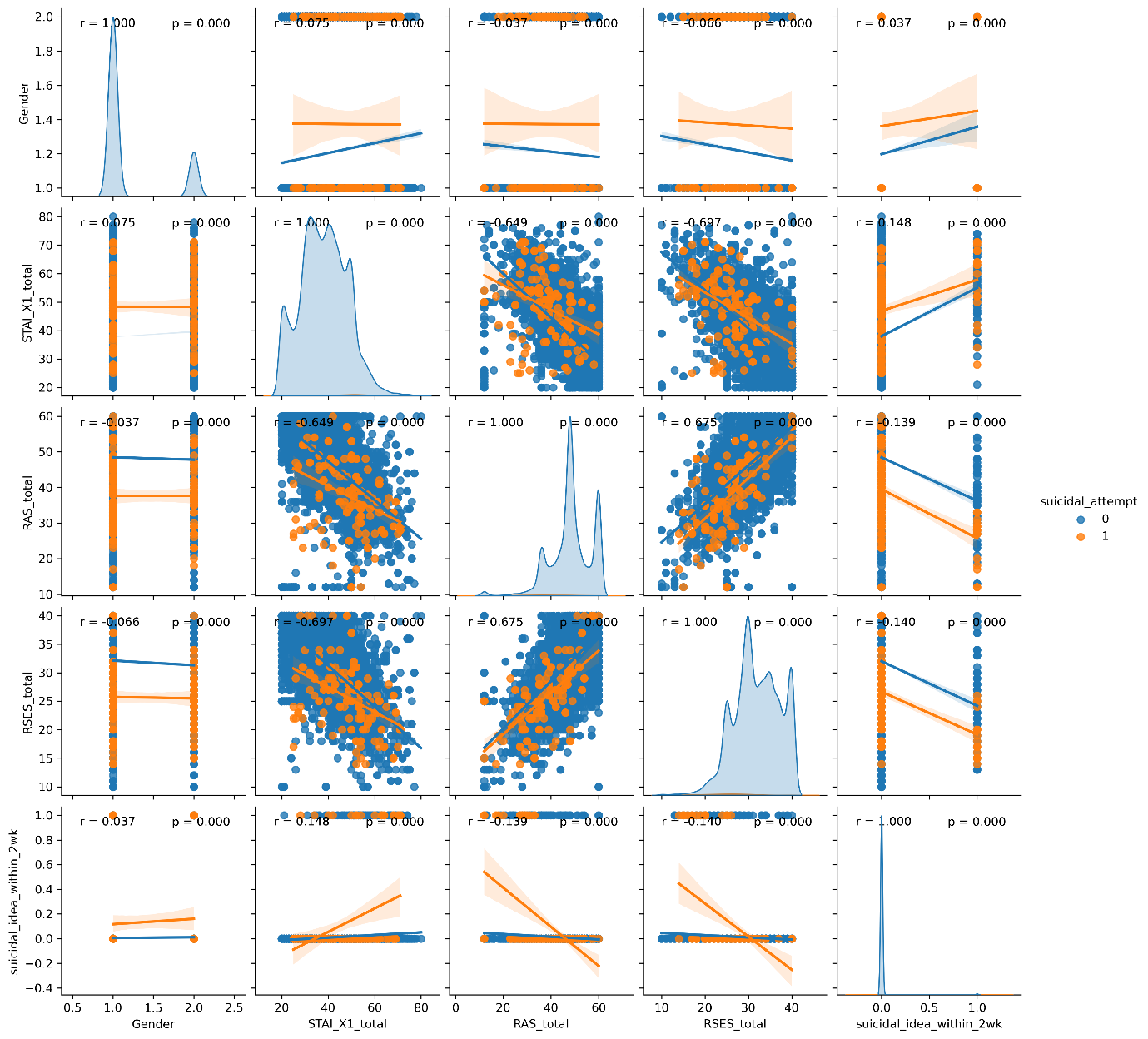


**Supplementary** **Figure 1.** Exploratory data analysis


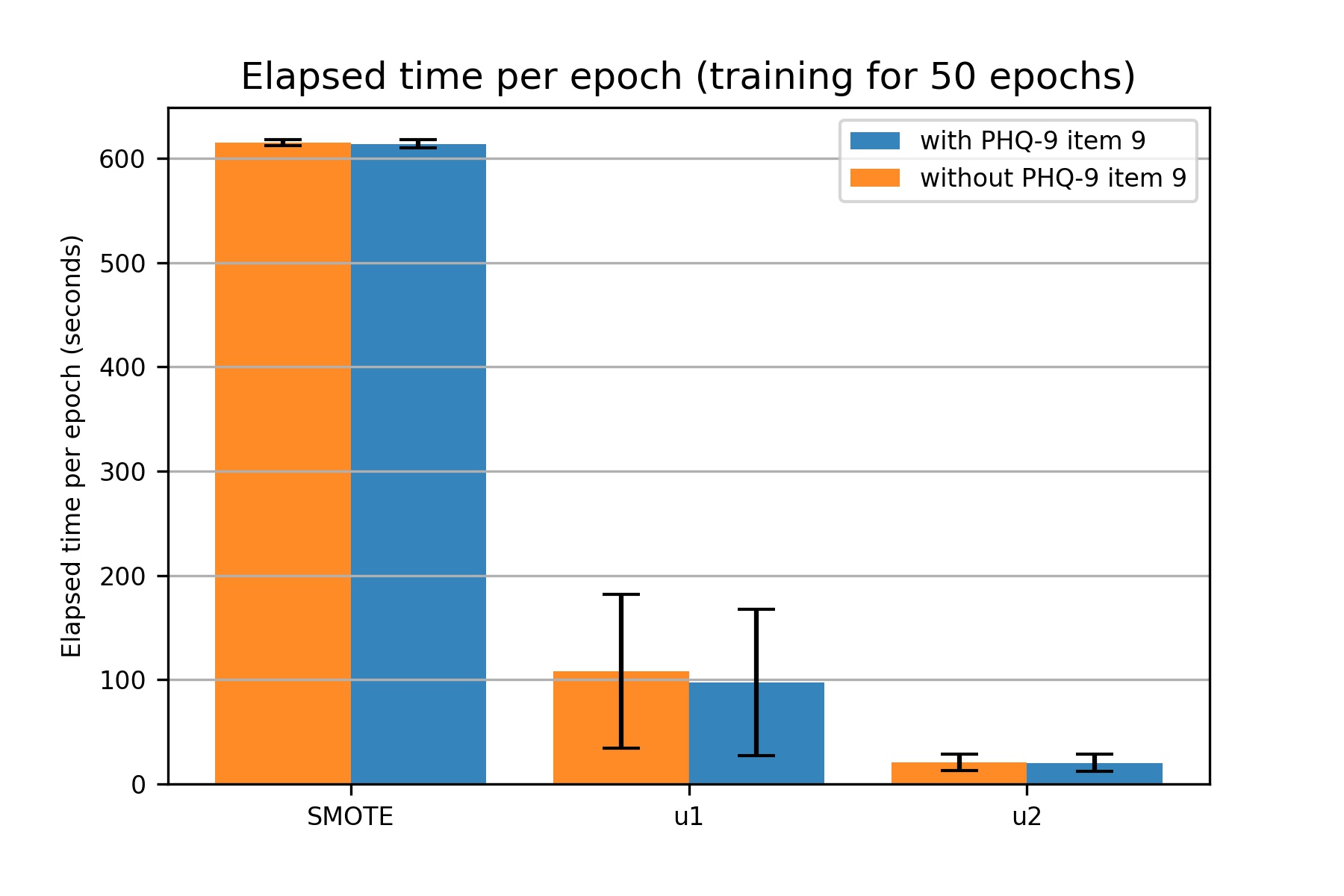


**Supplementary Figure 2.** Elapsed time per epoch for training. When using oversampling (SMOTE), the training time was the longest, with an average of about 10 minutes per epoch as the number of samples increased. For the undersampling, u1 and u2 took about 100 and 20 seconds on average, respectively. There were no significant differences when comparing with and without item 9 of the PHQ-9 scale for all sampling methods (p>0.23; via two sample independent t-test).

**
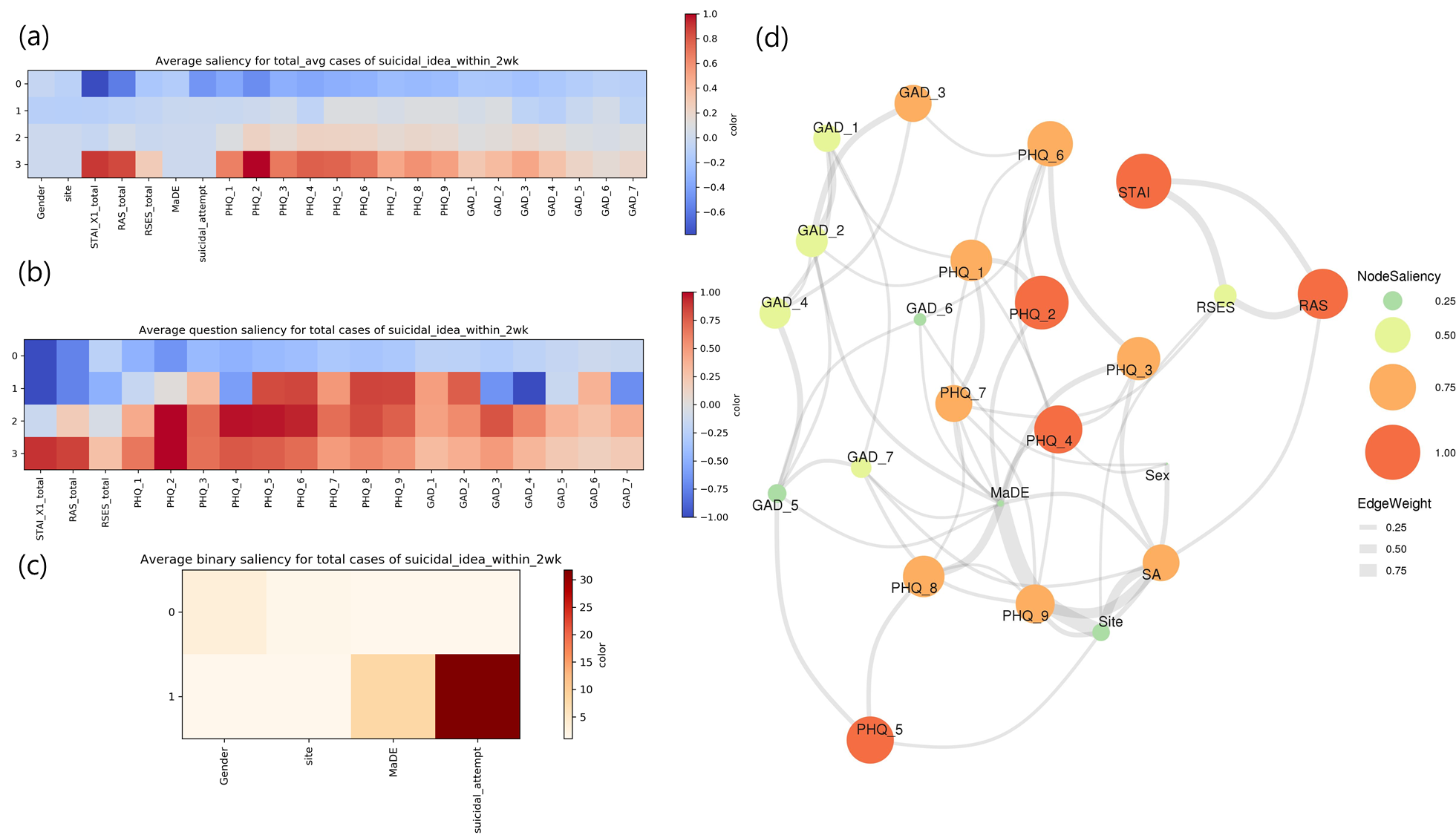
**

**Supplementary Figure 3.** Attention plots for training/validation set. (a) Raw averaged attention plots, (b) attention plot comparing questionnaire items, (c) attention plot comparing binary items, (d) attention plot for mixed Gaussian model-based graphical network (generated with the “mgm” R package for visualization) for questionnaire items in training/validation set (*n*=17,482).

**
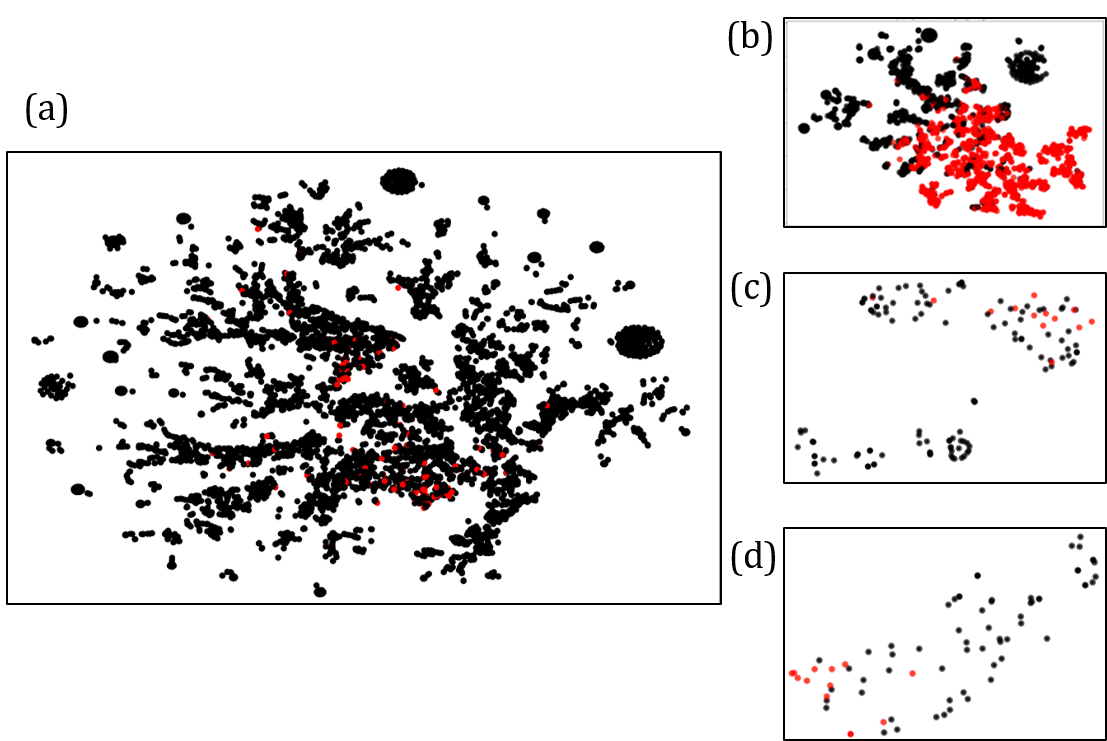
**

**Supplementary Figure 4.** T-distributed stochastic neighbor embedding (t-SNE) plots of (a) ensemble GIN model for test set (177 positive cases out of 14,238), (b-d) GIN-synthetic minority over-sampling technique (SMOTE) (1,734 positive cases out of 3,468), GIN-u1 (13 positive cases out of 142), and GIN-u2 (13 positive cases out of 78) for the validation set.

**Supplementary References**

1. Simon, G.E.*, et al.* Predicting suicide attempts and suicide deaths following outpatient visits using electronic health records. *American Journal of Psychiatry* **175**, 951-960 (2018).

2. Tibshirani, R. Regression shrinkage and selection via the lasso. *Journal of the Royal Statistical Society: Series B (Methodological)* **58**, 267-288 (1996).

3. Cortes, C. & Vapnik, V. Support-vector networks. *Machine learning* **20**, 273-297 (1995).

4. Kroenke, K., Spitzer, R.L. & Williams, J.B. The PHQ-9: validity of a brief depression severity measure. *J Gen Intern Med* **16**, 606-613 (2001).

5. Spitzer, R.L., Kroenke, K., Williams, J.B. & Lowe, B. A brief measure for assessing generalized anxiety disorder: the GAD-7. *Arch Intern Med* **166**, 1092-1097 (2006).

6. Hahn, D.-W. Korean adaptation of Spielberger's STAI (K-STAI). *Kor J Health Psychol* **1**, 1-14 (1996).

7. Spielberger, C. State-trait anxiety inventory. The Corsini encyclopedia of psychology. *Hoboken: Wiley* **1**(2010).

8. Johnson, J., Gooding, P.A., Wood, A.M. & Tarrier, N. Resilience as positive coping appraisals: Testing the schematic appraisals model of suicide (SAMS). *Behaviour Research and Therapy* **48**, 179-186 (2010).

9. Rosenberg, M. Rosenberg self-esteem scale (RSE). *Acceptance and commitment therapy. Measures package* **61**, 18 (1965).

10. Park, S.-J., Choi, H.-R., Choi, J.-H., Kim, K.-W. & Hong, J.-P. Reliability and validity of the Korean version of the Patient Health Questionnaire-9 (PHQ-9). *Anxiety and mood* **6**, 119-124 (2010).

11. Ahn, J.-K., Kim, Y. & Choi, K.-H. The psychometric properties and clinical utility of the Korean version of GAD-7 and GAD-2. *Frontiers in psychiatry* **10**, 127 (2019).

12. Jung, Y.E.*, et al.* The Korean version of the Connor–Davidson Resilience Scale: an extended validation. *Stress and Health* **28**, 319-326 (2012).

13. Bae, H.-N., Choi, S.-W., Yu, J.-C. & Lee, J.-S. Reliability and validity of the Korean version of the Rosenberg Self-Esteem Scale (K-RSES) in adult. *Mood Emot* **12**, 43-49 (2014).

14. Sheehan, D.V.*, et al.* The Mini-International Neuropsychiatric Interview (M.I.N.I.): the development and validation of a structured diagnostic psychiatric interview for DSM-IV and ICD-10. *J Clin Psychiatry* **59 Suppl 20**, 22-33;quiz 34-57 (1998).

15. Shim, G. & Jeong, B. Predicting Suicidal Ideation in College Students with Mental Health Screening Questionnaires. *Psychiatry Investig* **15**, 1037-1045 (2018).

16. Xu, K., Hu, W., Leskovec, J. & Jegelka, S. How powerful are graph neural networks? *arXiv preprint arXiv:1810.00826* (2018).

17. Chawla, N.V., Bowyer, K.W., Hall, L.O. & Kegelmeyer, W.P. SMOTE: Synthetic minority over-sampling technique. *Journal of Artificial Intelligence Research* **16**, 321-357 (2002).

18. Lemaître, G., Nogueira, F. & Aridas, C.K. Imbalanced-learn: A python toolbox to tackle the curse of imbalanced datasets in machine learning. *The Journal of Machine Learning Research* **18**, 559-563 (2017).

19. Srivastava, N., Hinton, G., Krizhevsky, A., Sutskever, I. & Salakhutdinov, R. Dropout: A Simple Way to Prevent Neural Networks from Overfitting. *Journal of Machine Learning Research* **15**, 1929-1958 (2014).

20. Kingma, D.P. & Ba, J. Adam: A method for stochastic optimization. *arXiv preprint arXiv:1412.6980* (2014).

21. Wang, M.*, et al.* Deep graph library: Towards efficient and scalable deep learning on graphs. arXiv 2019. *arXiv preprint cs.LG/1909.01315*.

22. Van der Maaten, L. & Hinton, G. Visualizing data using t-SNE. *Journal of machine learning research* **9**(2008).


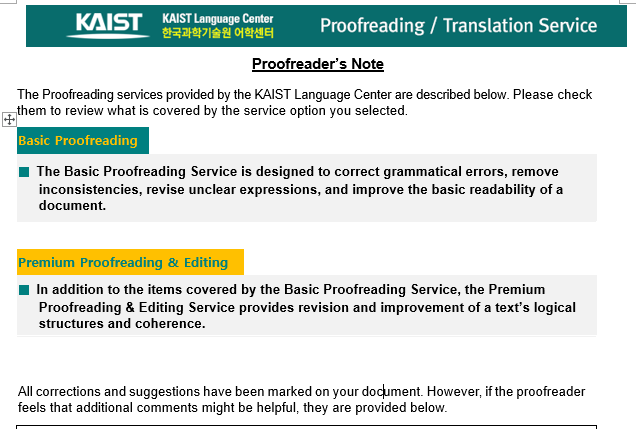


Hello,

I have left some balloon comments for you to check—there were a few places of concern. Otherwise, well-written paper. Best,.

- 1. Bruske
